# Unpacking excessive polypharmacy patterns among individuals living with chronic pain in Quebec: a longitudinal study

**DOI:** 10.3389/fpain.2025.1512878

**Published:** 2025-02-21

**Authors:** Gwenaelle De Clifford-Faugère, Hermine Lore Nguena Nguefack, Nancy Ménard, Sylvie Beaudoin, M. Gabrielle Pagé, Line Guénette, Catherine Hudon, Oumar Mallé Samb, Anaïs Lacasse

**Affiliations:** ^1^ Département des Sciences de la Santé, Université du Québec en Abitibi-Témiscamingue (UQAT), Rouyn-Noranda, QC, Canada; ^2^ Chaire de Recherche Institutionnelle en épidémiologie de la Douleur Chronique, UQAT, Rouyn-Noranda, QC, Canada; ^3^ Centre de Recherche, Centre Hospitalier de l’Université de Montréal, Montréal, QC, Canada; ^4^ Département d’anesthésiologie et de Médecine de la Douleur, Faculté de Médecine, Université de Montréal, Montréal, QC, Canada; ^5^ Axe Santé des Populations et Pratiques Optimales en Santé, Centre de Recherche du CHU de Québec—Université Laval, Québec, QC, Canada; ^6^ Faculté de Pharmacie, Université Laval, Québec, QC, Canada; ^7^ Département de Médecine de Famille et Médecine d’urgence, Faculté de Médecine et des Sciences de la Santé, Université de Sherbrooke, Sherbrooke, QC, Canada

**Keywords:** polypharmacy, chronic pain, healthcare, trajectories, group-based trajectory modelling, health administrative data

## Abstract

**Introduction:**

Excessive polypharmacy, which can be defined as the concurrent use of ≥10 medications, is prevalent among individuals with chronic pain. However, it remains unclear how it may vary between individuals or over time.

**Objectives:**

This study aimed to describe and identify factors associated with trajectories of excessive polypharmacy.

**Methods:**

A retrospective longitudinal study was conducted using the TorSaDE Cohort, which links Canadian Community Health Surveys (2007–2016) and Quebec health administrative databases. Among 9,156 adults living with chronic pain and covered by public prescribed drug insurance, the presence of excessive polypharmacy (yes/no) was assessed monthly for one-year post-survey completion (12 time points). Group-based trajectory modelling was applied to identify groups with similar patterns over time (trajectories). Multivariable multinomial regression was used to identify factors associated with trajectory membership.

**Results:**

Four trajectories were obtained: (1) “No excessive polypharmacy” (74.8%); (2) “Sometimes in excessive polypharmacy” (8.6%); (3) “Often in excessive polypharmacy” (6.1%); 4) “Always in excessive polypharmacy” (10.5%). Factors associated with the “always in excessive polypharmacy” trajectory membership were: being older, being born in Canada, having a lower income, having a higher comorbidity index score, more severe pain intensity, and more daily activities prevented by pain, reporting arthritis or back pain and poorer perceived general health, and having a family physician. Using opioids or benzodiazepines, having a lower alcohol consumption, doing less physical activity, a higher number of prescribers and visits to a family physician also predicted being always in excessive polypharmacy.

**Discussion:**

This study identifies distinct trajectories of excessive polypharmacy in adults with chronic pain, emphasizing key sociodemographic and clinical factors and the need for tailored interventions for effective medication management.

## Introduction

Chronic pain (CP) can be defined as pain persisting or recurring for more than three months (1) and affects 20% of adults worldwide (2). Despite decades of research on CP, it remains poorly recognized, misdiagnosed, and its management is often suboptimal (3–9). Considering the impact of CP at the individual and societal levels (9), it is important to harness real-world data and gain a deeper understanding of the context in which treatments are prescribed and used, and examining the outcomes. One of the areas of interest lies in the significant proportion of individuals living with CP who use medications, even though various physical and psychological treatment approaches should be employed (8). In fact, most individuals living with CP (62%–94%) use medications for pain management (10–13) and are often simultaneously treated with medications from different therapeutic classes (e.g., opioids, antidepressants, anti-inflammatories, anticonvulsants, acetaminophen, prescribed cannabinoids) (14, 15). Furthermore, medications used for various comorbidities add to the count (16). Polypharmacy, defined as the concurrent use of multiple medications, is thus the rule rather than the exception in CP (17–19).

The most commonly used definition of polypharmacy (20–22) and endorsed by the World Health Organization (23) is the concomitant use of 5 medications or more. Polypharmacy is considered excessive when 10 or more medications are used. The presence of pain is a known determinant of polypharmacy (24–26). A recent Canadian study highlighted that 7 out of 10 adults living with CP were in a state of polypharmacy, and 1 out of 4 was in excessive polypharmacy (18). Using as many medications can lead to significant risks for the person, such as drug interactions (27, 28) and drug cascades (29). Also, polypharmacy is correlated with higher mortality rates (30, 31), depression (32), lower quality of life (33) and institutionalization (31). Yet, literature suggests that polypharmacy can be rational and can lead to positive clinical outcomes by approaching diseases through multiple mechanisms of action (21, 34). For example, in a context where opioid prescriptions are highly debated for chronic non-cancer pain and where patients often experience forced withdrawal and inadequate pain relief (35), rational polypharmacy potentially offers an alternative strategy by integrating non-opioid medications and addressing the multifaceted aspects of CP. Besides patient's condition, medication use and polypharmacy can be shaped by healthcare structure (universal vs. privatized) which influence access to care, prescription practices, and coordination among prescribers (36, 37).

The likelihood of experiencing excessive polypharmacy may vary between groups of individuals (inter-individual variability) and over time (intra-individual variability). It is therefore possible to identify clinical situations that are potentially favourable or unfavourable (e.g., groups of individuals in which the likelihood of experiencing excessive polypharmacy remains relatively low over time or remains high over time). Person-centred statistical approaches such as latent class analysis offer the opportunity to group together individuals with similar trajectories of excessive polypharmacy over time and to identify specific sub-populations at need for better healthcare support (38). To date, although such approaches have been employed to better understand polypharmacy trajectories (31, 39), to our knowledge, they have never been applied within a population of individuals living with CP. In order to answer the research questions “What are the different profiles of excessive polypharmacy of individuals living with CP in the community?” and “Is excessive polypharmacy a stable phenomenon over time or does it occur intermittently?”, this study aimed to: (1) model and describe the trajectories of excessive polypharmacy (identification of groups with similar patterns of excessive polypharmacy over time), and (2) identify factors associated with trajectory memberships.

## Methods

### Study design and data sources

A longitudinal study was conducted using existing data from the TorSaDE Cohort (40), a sample of 102,148 participants which links five cycles of Statistics Canada's Canadian Community Health Survey (CCHS; 2007–2008, 2009–2010, 2011–2012, 2013–2014 and 2015–2016 cross-sectional questionnaires) with the Quebec health administrative databases (1996 to 2016 longitudinal healthcare and pharmaceutical services databases). This cohort presents the opportunity to study the use of healthcare and medications in a probabilistic community sample of participants, while benefiting from the strength of self-reported data combined with longitudinal health administrative data (40). In fact, individuals living with CP are difficult to identify with administrative data alone (41, 42).

#### Canadian community health survey

The CCHS collects health data on a representative sample of Canadians aged 12 and older (probability sampling) (43). Indigenous individuals living on reserves, full-time members of the Canadian Armed Forces, institutionalized individuals, or residents of Nunavik and Terre-Cries-de-la-Baie-James (together 3% of Canadians) are not included. Several questions have demonstrated test-retest reliability (44), and high response rates are observed [69.8–78.9%, depending on cycles (45)]. Survey participants gave informed consent to Statistics Canada for the linkage of their responses to provincial health administrative databases and use for research purposes when they take part in the survey.

#### Quebec health administrative databases

In the Quebec province, the population is covered by a provincial universal health insurance program administered by the *Régie de l'assurance maladie du Québec* (RAMQ) (46). The health insurance covers the cost of medical visits, emergency department visits, hospitalizations and procedures offered to all residents (8 million people). For prescribed drugs, only a portion of the population is covered: (1) people who are not eligible for private drug insurance with their employer or their spouse's employer, (2) who are ≥65 years old, or (3) receiving last-resort financial assistance. These groups represent approximately 45% of the population (47). Although this may lead to a sample that is older or socioeconomically disadvantaged, our goal was to capture a broader population that reflects the diversity of individuals covered by public insurance, beyond just those aged ≥65. Restricting the analysis to those ≥65 would limit the generalizability of our findings to younger adults who are also at risk for excessive polypharmacy. The pharmaceutical services database contains detailed information on the dispensing of prescribed drugs covered by the public plan, and the validity of its contents has been demonstrated (48).

For healthcare services research, the TorSaDE Cohort is a unique database in Canada that contains a variety of sociodemographic variables not found in health administrative databases. It has been described in detail elsewhere (40). A secure virtual server provided by the Quebec Statistical Institute holds de-identified TorSaDE data. The project was approved by relevant research ethics boards: (1) Université du Québec en Abitibi-Témiscamingue (# 2018-02—Lacasse, A.), (2) Centre hospitalier universitaire de Sherbrooke (#2017-1504), (3) *Commission d'accès à l'information du Québec* (#1013990). In terms of patient engagement, two persons with lived experience of CP (NM and SB), were members of the team and participated in the conceptualization of the project and interpretation of results.

### Study population

**Figure 1:**
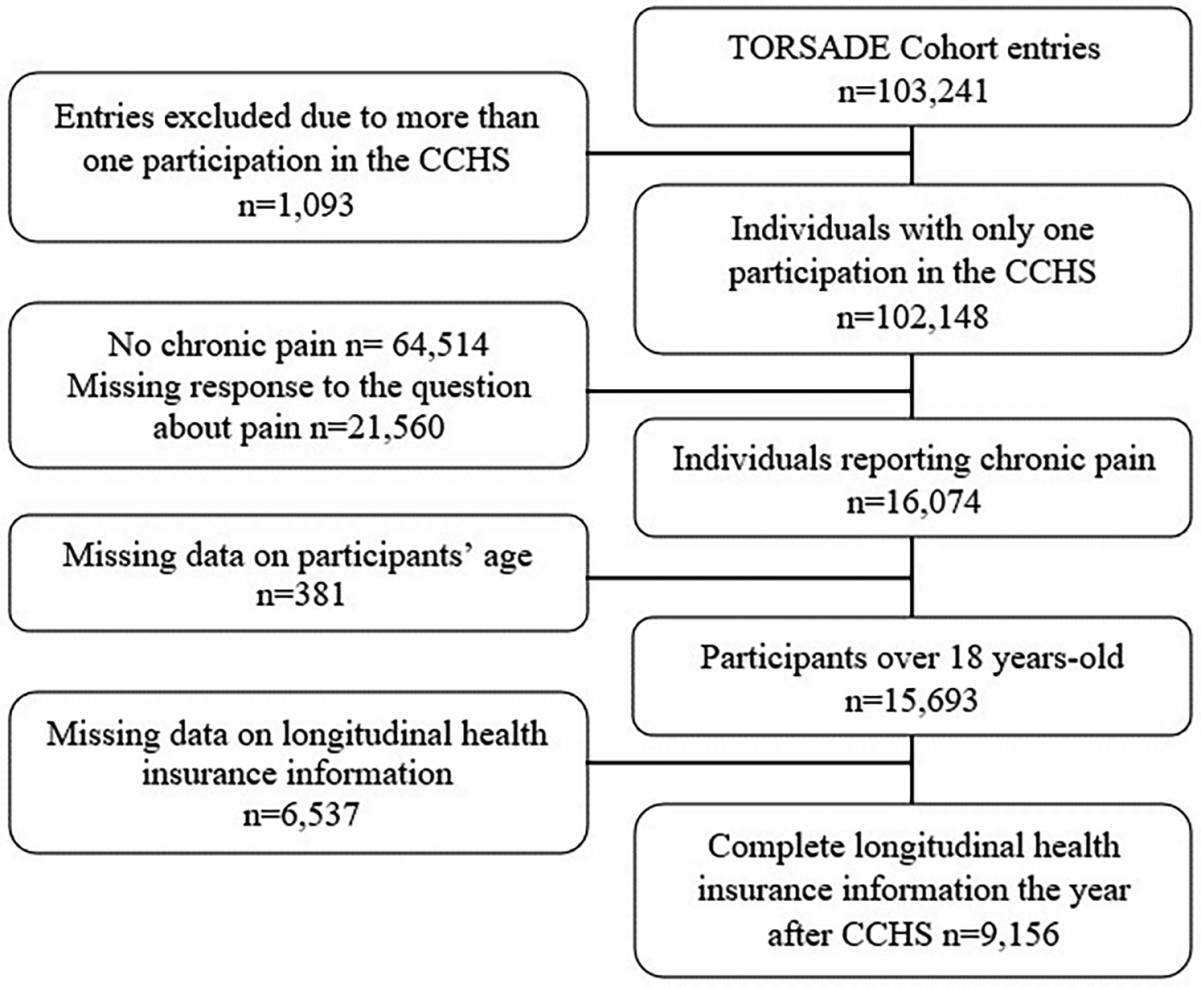
Flowchart. CCHS, Canadian community health survey.

As shown in Figure 1, the sample was constituted by applying four inclusion criteria to TorSaDE's participants (*n* = 102,148): (1) having only one participation in the survey (only the most recent Canadian Community Health Survey entry was retained for participants with more than one entry); (2) reporting CP (answering “No” to the question, “*Are you usually free of pain or discomfort?*”). Even though it differs from commonly used definitions of chronicity based on symptoms' duration (1, 49–51), such a definition provides prevalence estimates comparable to those obtained with more conventional definitions (2) and many highly cited CP epidemiology studies have used this definition (52–58); **(3)** be at least 18 years old; and **(4)** being covered by the public drug plan for the year following survey completion (the index date was defined as the date of completion of the survey). Thus, among the 102,148 TorSaDE participants, 9,156 were selected.

### Study variables

#### Excessive polypharmacy trajectories

Excessive polypharmacy was defined as the concomitant use of ≥10 medications (all indications included). The threshold of 10 medications is widely recognized in clinical practice and research as a marker for increased risks of drug interactions, side effects, and management challenges (21, 59, 60). While categorizing the outcome does reduce some information compared to using a continuous variable, it enhances the interpretability of our findings for clinicians and policymakers, especially in identifying risk factors and guiding interventions for excessive polypharmacy. As the pharmaceutical services database contains detailed information on calendar dates prescriptions were filled, monthly dichotomous measures of excessive polypharmacy (yes or no) could be calculated for each participant (12 repeated measures; periods of 30 days). A 1-year post-index date time window was chosen because it maximized the chances of capturing temporal variations in excessive polypharmacy trajectories and ensured that the sociodemographic and clinical profile captured in the survey (potential predictors) did not change significantly. Repeated measures of excessive polypharmacy were modelled into trajectories using group-based trajectory modelling (GBTM) (38, 61–64), a statistical approach grouping participants with similar patterns of outcomes over time and allowing the discovery of hidden subgroups within the data without the need to set arbitrary classification thresholds (see full description in the statistical analysis section). Trajectory group membership was then used as a categorical dependent variable.

#### Factors associated with excessive polypharmacy & covariables

The TorSaDE Cohort includes thousands of variables, requiring a selection of sociodemographic and clinical variables relevant to CP and its treatment. As we aimed to identify factors associated with excessive polypharmacy trajectory membership, the choice of variables to be considered was based on the Andersen model of health services use (65), widely used in healthcare (66) and drug utilization (67) studies. Andersen's model (65) postulates that healthcare utilization (in our case prescriptions) is influenced by predisposing factors (e.g., age, sex, country of birth), enabling factors (e.g., household income, access to a pain clinic), and need factors (e.g., pain intensity, activities prevented by pain, perceived general health). In this study, the following variables were thus included: (1) Sociodemographic variables: Age, sex at birth, self-identified race (white or others), indigenous self-identification (yes or no), country of birth (Canada or others), education level, relationship status (in or not in a relationship), annual household income (Can$), number of people in the household, living in a remote region (yes or no; based on Quebec Revenue Agency classification), living in a rural area (yes or no; based on the postal code second character); (2) Pain-related variables: Pain intensity (mild, moderate or severe), pain interference (none, few, some or most daily activities prevented by pain or discomfort), self-reported back pain (yes or no), self-reported arthritis (yes or no), opioid use in the 30 days following survey completion (yes or no), benzodiazepine use in the 30 days following survey completion (yes or no); (3) General health and lifestyle profile variables: Combined comorbidity index of Charlson and Elixhauser (68) computed in the year before survey completion (taking into consideration a variety of comorbidities other than pain), perceived general health and mental health (excellent, very good, good or fair, bad), alcohol consumption in the past 12 months (regular, occasional, not drinking), smoking (regular, occasional, not smoking), physical activity (regular, occasional, rare), lifestyle (active, moderately active, inactive), and (4) Healthcare related-variables: Visiting a pain clinic in the year before survey completion (yes or no; medical visits associated with a 4X1 center code or professional services billed for services rendered in a pain clinic (i.e., anesthesia services coded 41,055, 41,056, 41,057, 41,058 and 41,059), reporting having a family physician (yes or no), in addition to the past-year number of hospitalizations, visits to a family physician, visits to a specialist, emergency room visits, prescribers consulted, and different physicians consulted.

### Statistical analysis

Descriptive statistics were used to characterize participants (means and standard deviations for continuous variables and numbers and proportions for categorical variables). The polypharmacy profile of participants was also described for various time windows (proportion of individuals using ≥5 or ≥10 medications; mean number of medications used). Excessive polypharmacy trajectories were modelled using a latent class modelling approach (38). Among the different modelling approaches, we chose group-based trajectory modelling (GBTM) due to the longitudinal study design and the repeated measures of a categorical variable over time, i.e., excessive polypharmacy (yes/no) (38, 62). GBTM is a statistical approach allowing to group participants with similar patterns of variables over time (38, 61–64). The modelling of trajectories was performed for 1, 2, 3, 4 and 5 trajectory groups and different curve possibilities (linear, quadratic, and cubic) for each trajectory. Different models were tested and two criteria were used for selecting the best one: (1) Lowest Bayesian information criterion (BIC; absolute value) and (2) Minimum of 5% of participants per group (38). We also ensured that the model identified using the two criteria above was clinically interpretable (38), e.g., a classification that is sufficiently discriminative and provides a logical label for each group. See Supplementary Material S1 for model fit indices. GBTM was applied in the whole study sample and then stratified by sex at birth to assess differences of excessive polypharmacy trajectories in males and females, according to best practices for better integration of sex and gender in research (69). Next, the profile of participants according to their trajectory membership (subgroups) was depicted and the differences between the trajectories were determined by Chi-square for each variable. Finally, a multivariable multinomial regression model was used to identify factors (independent variables) associated with trajectory membership (dependent variable). Results are presented as adjusted odds ratios (OR) and 95% confidence intervals (95% CI). Based on the Andersen model (65), an *a priori* selection of variables was made (all the variables listed above were included in our multivariable model). Indeed, the goal was to identify all associated variables rather than to construct a predictive model. Due to our substantial sample size, this method was favoured over criticized selection techniques such as relying on bivariate regression analysis *p*-values (70) or stepwise selection (71). All variables considered in the model are detailed in Supplementary Material S2 and S3. Multicollinearity was tested according to variance inflation factors (VIFs), who were below 3.0 for all variables included in the multivariable models (72). Goodness of fit of the model was tested using the Hosmer-Lemeshow test. No multiple imputation was applied as missing data proportion was low across variables of interest (≤4.3%). A *p*-value of 0.05 was used as the cutoff for statistical significance. All statistical analyses were performed using SAS® version 9.4 (SAS Institute, Cary, NC, USA).

## Results

Analyses were conducted among 9,156 adults living with CP and covered by the public drug insurance plan in the year following survey completion (Figure 1). Characteristics of participants are presented in Table 1. The mean age was 63.5 years (±15.3) with a range of 18 to 101 years. The majority of our sample was female (64.5%), born in Canada (82.9%) and self-identified as White (93.5%). Most had moderate (53.7%) or severe (25.3%) pain intensity, as well as none (33.9%) or a few (33.0%) daily activities prevented by pain or discomfort. Moreover, 42.7% of our sample self-reported back pain and 48.4% self-reported arthritis (non-mutually exclusive variables). The polypharmacy profile of participants is described in Table 2. Prevalence of polypharmacy (≥5 medications) and excessive pharmacy (≥10 medications) in the 30 days following survey completion was respectively 48.3% and 16.3%.

**Table 1:** Sample characteristics.

Characteristics* (*n* = 9,156)	No. (%) of participants**	
Sociodemographic profile		
Age (years)		
18–24	175	(1.91)
25–44	910	(9.94)
45–64	3,110	(33.97)
65–79	3,736	(40.80)
≥80	1,225	(13.38)
Sex at birth		
Females	5,908	(64.53)
Males	3,248	(35.47)
White self-identified race		
Yes	8,563	(93.52)
Indigenous self-identification		
Yes	228	(2.49)
Country of birth		
Canada	8,503	(92.87)
Other	650	(7.10)
Education level		
No secondary diploma	3,751	(40.97)
Secondary diploma	1,171	(12.79)
College diploma/Registered apprenticeship or other trades certificate or diploma	3,077	(33.61)
University diploma	1,032	(11.27)
Relationship status		
In a relationship	3,847	(42.02)
Not in a relationship	5,304	(57.93)
Annual household income (Can$)		
<20,000	3,083	(33.67)
20,000–39,999	3,482	(38.03)
40,000–59,999	1,468	(16.03)
60,000–79,999	612	(6.68)
≥80,000	511	(5.58)
Number of people in the household—mean ± SD	1.69	±0.92
Living in a remote region		
Yes	2,448	(26.74)
No	6,708	(73.26)
Living in a rural area		
Yes	2,898	(31.65)
No	6,258	(68.35)
Pain symptoms		
Pain intensity		
Mild	1,836	(20.05)
Moderate	4,913	(53.66)
Severe	2,314	(25.27)
Activities prevented by pain or discomfort		
None	3,101	(33.87)
A few	3,018	(32.96)
Some	1,531	(16.72)
Most	1,457	(15.91)
Self-reported back pain (except fibromyalgia and arthritis)		
Yes	3,909	(42.69)
Self-reported arthritis (except fibromyalgia)		
Yes	4,435	(48.44)

SD, standard deviation.

* Proportion of missing data across presented variable ranged between 0.03 and 4.43%. Listwise deletion was applied for the subsequent analyses.

** Unless stated otherwise.

**Table 2:** Polypharmacy profile.

*n* = 9,156	During the 30 days following survey completion	During the 60 days following survey completion	During the 90 days following survey completion	During the 365 days following survey completion
	*n* (%)	*n* (%)	*n* (%)	*n* (%)
Polypharmacy (≥5 medications)	4,426 (48.34)	5,079 (55.47)	5,401 (58.99)	6,766 (73.90)
Excessive polypharmacy (≥10 medications)	1,492 (16.30)	1,957 (21.37)	2,285 (24.96)	4,017 (43.87)
	Mean ± SD (range)	Mean ± SD (range)	Mean ± SD (range)	Mean ± SD (range)
Number of different prescribed medications (all indications combined)	5.10 ± 4.49 (0–29)	5.94 ± 4.84 (0–34)	6.44 ± 5.13 (0–49)	9.45 ± 6.88 (0–49)

SD, standard deviation.

### Modelling trajectories of excessive polypharmacy

**Figure 2:**
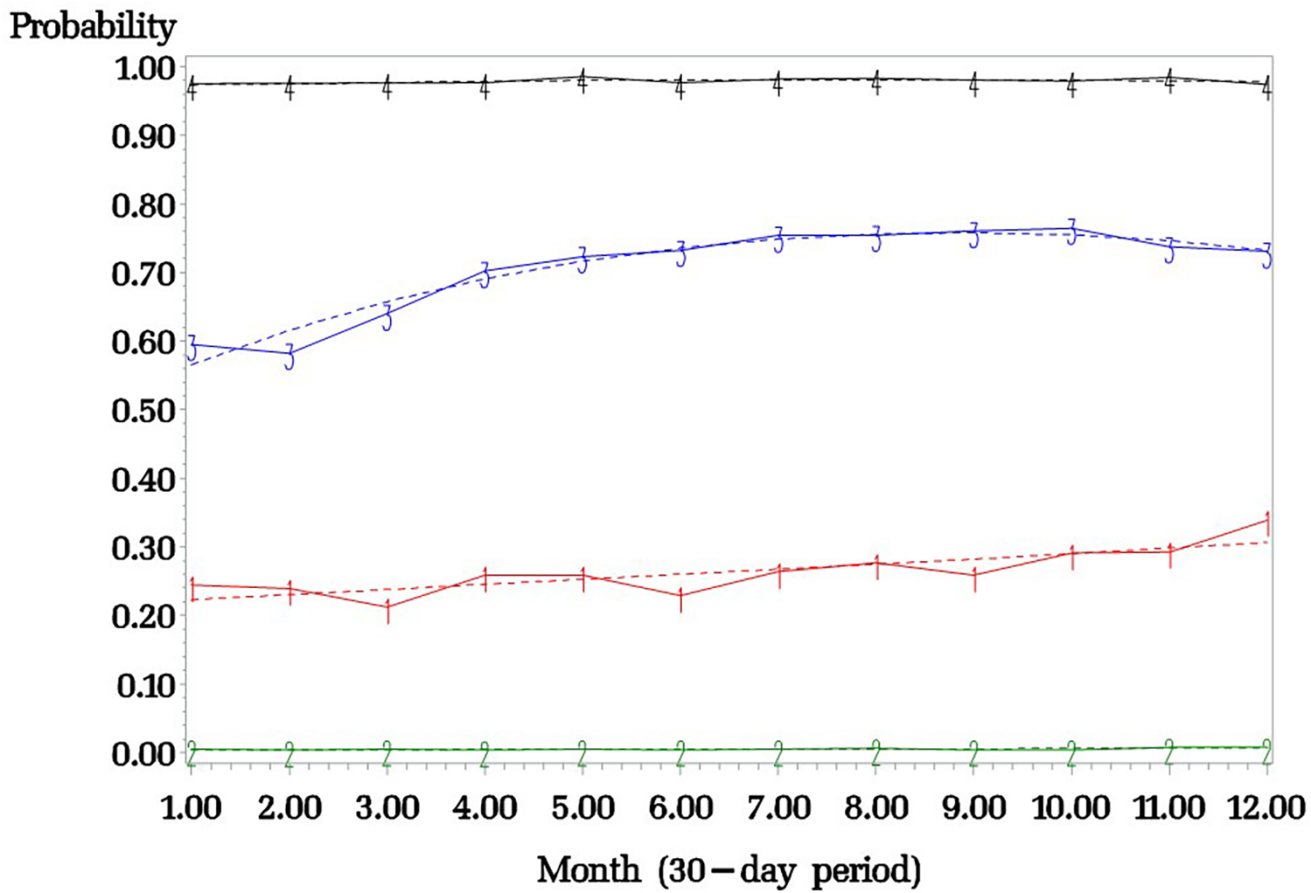
Trajectories of excessive polypharmacy in the whole sample. Black curve: “always in polypharmacy” group; Blue curve: “often in polypharmacy” group; Red curve: “sometimes in polypharmacy” group; Green curve: “no excessive polypharmacy” group. Plain line: Observed prevalence of excessive polypharmacy. Interrupted line: Estimated prevalence of excessive polypharmacy by the group-based trajectory modelling.

A four-trajectory model was retained (Figure 2): (1) Persons who never experience excessive polypharmacy (74.8% of the sample; label: “no excessive polypharmacy” group; Green curve); (2) Persons with a relatively moderate likelihood of experiencing excessive polypharmacy over time with a slight increase (linear shape positive) in the likelihood of having excessive polypharmacy during the study period (8.6% of the sample; label: “sometimes in excessive polypharmacy” group; Red curve); (3) Persons with a relatively high likelihood of experiencing excessive polypharmacy over time with a slight increase (linear shape positive and quadratic shape negative) in the likelihood of having excessive polypharmacy during the study period (6.1% of the sample; label: “often in excessive polypharmacy” group; Blue curve); (4) Persons consistently in an excessive polypharmacy status (10.5% of the sample; label: “always in excessive polypharmacy” group; Black curve).

**Figure 3:**
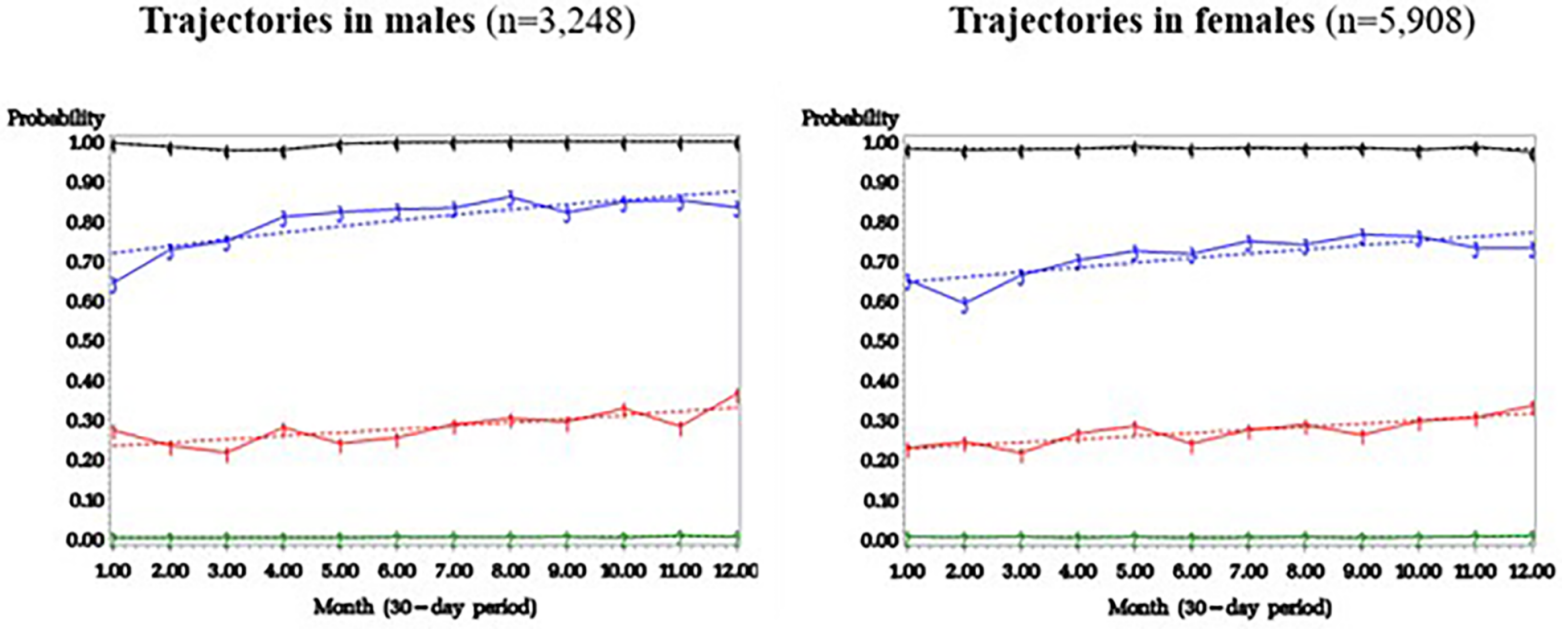
Trajectories of excessive polypharmacy in males and in females. Plain line: Observed prevalence of excessive polypharmacy. Interrupted line: Estimated prevalence of excessive polypharmacy by the group-based trajectory modelling.

Stratification of trajectory models was performed by sex at birth (males and females). When GBTM was applied among males (*n* = 3,248) and females (*n* = 5,908), a four-trajectory model was retained for both groups (Figure 3), with curves similar to those found in the whole sample: (1) “No excessive polypharmacy” group (79.9% of males and 73.6% of females); (2) “Sometimes in excessive polypharmacy” group (6.7% of males and 8.8% of females); (3) “Often in excessive polypharmacy” group (6.7% of males and 6.1% of females); (4) “Always in excessive polypharmacy” group (6.7% of males and 11.5% of females). See Supplementary Material S1 for model fit indices for males and for females.

### Factors associated with trajectory membership

**Figure 4:**
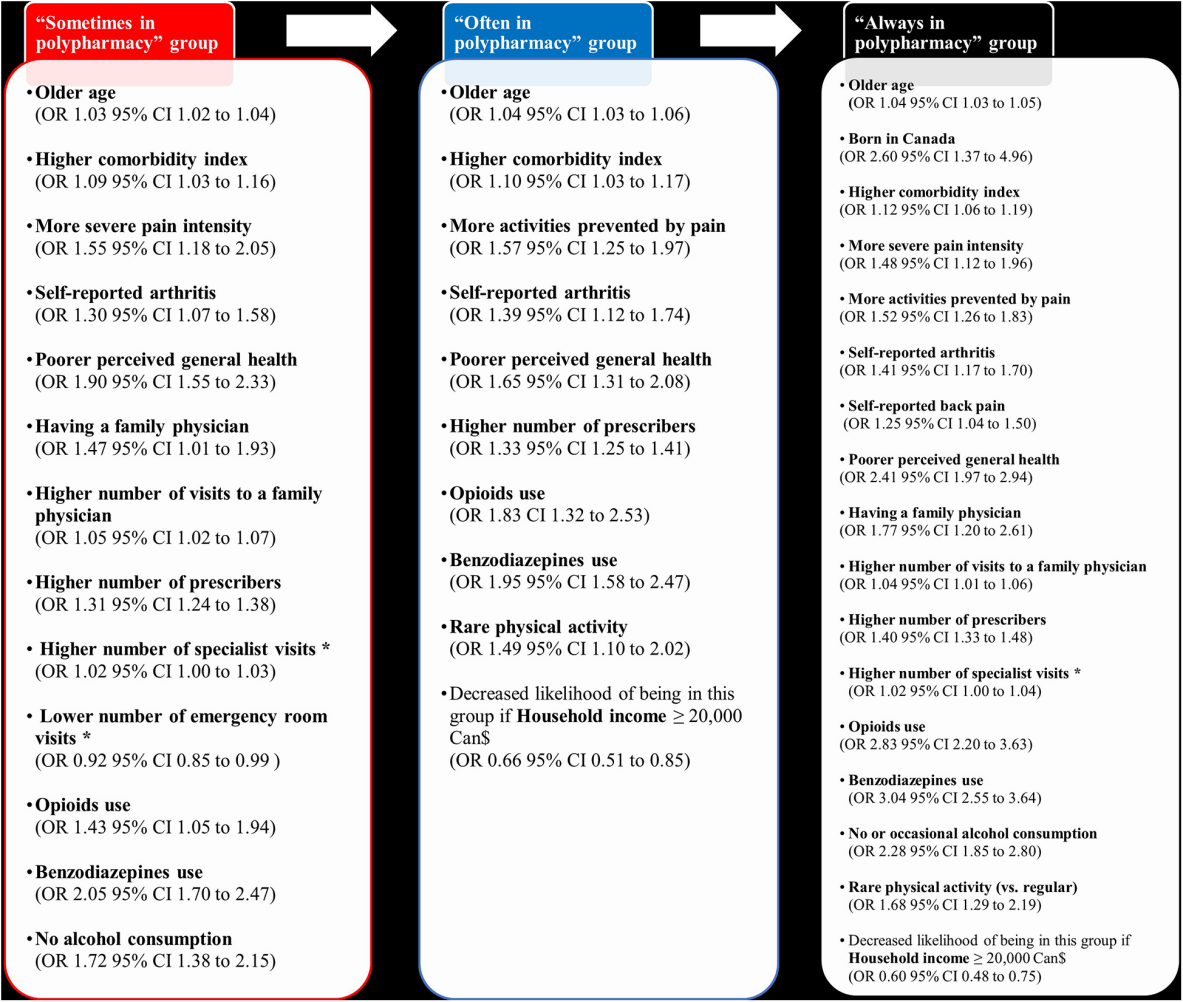
Participants’ sociodemographic and clinical characteristics significantly associated with the different trajectories in the multivariable multinomial regression analysis (reference: “no polypharmacy group”). Age, the number of people in the household, the comorbidity index of Charlson and Elixhauser, and the number of healthcare visits or prescribers were included in the model as continuous variables; the remaining of variables were categorical. *Borderline statistical significance.

Multivariable multinomial regression model was used to identify participants' sociodemographic and clinical characteristics associated with each trajectory while controlling for confounding. Figure 4 illustrates statistically significant factors associated with each trajectory group as compared to the “no excessive polypharmacy” group. For the reader's benefit, a comprehensive description of each group profile, along with bivariate comparisons, is provided in Supplementary Material S2. The complete multivariable model results can be found in Supplementary Material S3. The most important predictors of various excessive polypharmacy trajectories (adjusted OR ≥ 1.5) were: more severe pain intensity and activities prevented by pain, poorer perceived general health, opioids and benzodiazepines use, less alcohol consumption, being born in Canada, having a family physician, and rare physical activity (reference: regular physical activity). Being older, having a high comorbidity index, self-reporting arthritis, a poor perceived general health, a high number of prescribers, using opioids and benzodiazepines were common predictors of the three excessive polypharmacy trajectories.

## Discussion

This study aimed to model the trajectories of excessive polypharmacy (≥10 medications) in a large sample of adults living with CP. Four patterns of excessive polypharmacy emerged: (1) “No excessive polypharmacy” group; (2) “Sometimes in polypharmacy” group; (3) “Often in excessive polypharmacy” group; (4) “Always in excessive polypharmacy” group. Our results thus offer confirmation that different profiles of excessive polypharmacy exist within the community and that excessive polypharmacy is not necessarily a stable phenomenon over time (for many it occurs intermittently). Importantly, it is still 16.3% of individuals living with CP (1 out of 6) who are in a state of excessive polypharmacy when looking at 1-month data.

### Prevalence of polypharmacy

Among individuals living with CP, the literature suggests prevalence estimates of polypharmacy (≥5 medications) ranging from 19% to 89%, and from 5% to 49% for excessive polypharmacy (≥10 medications) depending on the country, the sample characteristics, the definition of polypharmacy, and the type of medications considered (17–19, 73–77). The most comparable study is Zahlan et al. (18) in terms of country, sample and definition of polypharmacy. Our 1-month prevalence estimates were, however, lower than those found by Zahlan et al. (18) (polypharmacy: 48.3 vs. 71.4%; excessive polypharmacy 16.3% vs. 25.9%), possibly as our study accounted only for prescribed drugs (vs. Zahlan et al. study also considered over-the-counter medications).

### Sex and polypharmacy

For both male and female participants, we found four similar excessive polypharmacy trajectories. One difference concerned the proportion of participants per trajectory: slightly more males than females were in the “no excessive polypharmacy” group (80% vs. 74%), and that slightly more females than males were in the “always in excessive polypharmacy” group (12% vs. 7%). This result is consistent with previous studies showing that polypharmacy is more frequent in females than in males (the terms sex and gender were used interchangeably in these studies) (78–82). When Zahlan et al. (18) assessed factors associated with self-reported use of ≥10 medications, gender identity was not significantly associated with excessive polypharmacy according to the multivariable analysis. Also, no statistical difference across sex was found by Giummara et al. (19) as for the prevalence of polypharmacy in a CP sample (defined as ≥2 pain medications). According to a recent systematic review of factors associated with polypharmacy including 106 studies (not in the field of pain) (83), no association was highlighted between sex and polypharmacy. Future studies should aim at achieving a thorough sex- and gender-based analysis to further investigate such differences, including various ways of operationalizing polypharmacy (e.g., ≥5 medications, ≥10 medications, number of medications and polypharmacy longitudinal trajectories).

### Age and excessive polypharmacy

Many pain and non-pain studies showed a positive association between older age and polypharmacy (18, 75, 83–85). The increase in the population aged 65 and over (86, 87) as well as the lengthening of life expectancy, raises methodological considerations for research such as the need to consider sub-populations aged 80 and over. According to the World Health Organization (88), 1 out of 6 people in 2030 will be aged over 60 years old and the number of people aged over 80 will triple by 2050 to 426 million. That is why it is important to focus on people who are aging well. In this study describing four excessive polypharmacy trajectories, age was considered as a categorical variable to better understand the nuances between people over 65 and those over 80. Almost half the people in the “no excessive polypharmacy” trajectory were over 65, and 11% were over 80. This finding should be the subject of subsequent studies to better understand the characteristics of these “healthy” elderly people living with CP, in order to generate knowledge that fits in with government strategies on aging (87).

### Unexpected associations

Many associations found in this study can be explained by the increase in chronic diseases with age, thus the growing use of multiple medications. However, some associations were unexpected, such as a higher likelihood of being in excessive polypharmacy when not consuming alcohol or when born in Canada. Regarding alcohol, a possible explanation could be that it is not advisable to consume it when taking medication. As for the country of birth, our results align with a recent cross-sectional study that noted such an association (18). Potential explanations include differences in medication use between countries, lack of access to healthcare/prescribers or choice of using more traditional treatments (e.g., herbal therapy, acupuncture) (89, 90). Future research should be carried out to better understand these associations.

### Implications for clinicians

Persons living with CP are particularly at greater likelihood of polypharmacy (≥5 medications) and excessive polypharmacy (≥10 medications), as shown by previous studies (24–26) as well as ours. Although polypharmacy can be rational and can lead to positive clinical outcomes by approaching diseases through multiple mechanisms of action (21, 34), clinicians such as physicians, pharmacists and primary care nurses should be vigilant to excessive polypharmacy in CP and risk of adverse events. In fact, we found that 1 out of 6 persons living with CP was in the “often” or “always excessive polypharmacy” groups. Our results identified factors associated with trajectory membership, to guide clinicians in identifying those most at risk and offer a better follow-up. These people are older individuals, born in Canada, with a low annual household income, have a high comorbidity index, severe pain intensity, report higher functional impact of pain, report arthritis or back pain, have poorer general health, use opioids or benzodiazepines, and rarely engage in physical activity. Those individuals had a family physician, a high number of visits to a family physician, and a high number of prescribers, which could suggest that they may have the opportunity to receive a close medication follow-up, but could also suggest lack of coordinated care. Regular structured medication reviews (91) should be carried out in this population. Medication reviews seek to enhance a patient's understanding of their medication regimen, identify, and resolve drug therapy problems, and improve health outcomes (91). It can help clinicians inform persons living with CP of the risks associated with excessive polypharmacy, make them aware of the risks of drug interactions, optimize their pharmacotherapy and evaluate the relevance of deprescribing (92), especially as our results show that excessive polypharmacy is more frequent among opioids and benzodiazepines users. A possible explanation for the association between opioids use and polypharmacy could be a higher pain intensity (14), requiring more medications. However, adverse effects of opioids and benzodiazepines could affect daily activities (14). Future studies should explore patients' perspectives on the pros and cons of excessive polypharmacy, in addition to examining the impacts of interventions that guide patients toward potentially safer medication use patterns.

### Strengths and limits

The large sample size and diversity of profiles (community sample) contribute to the external validity of this study. However, including only participants with public drug insurance may lead to a sample that is older or socioeconomically disadvantaged, which could introduce bias. Also, it limited our ability to explore the impact of public vs. private coverage of prescription drugs on polypharmacy patterns. This should be explored in future studies. We must also emphasize that further studies should be conducted with more culturally diverse populations, considering the small number of Indigenous and racialized individuals in our sample. In terms of information bias, although we had access to the prescriptions claimed by each participant, a limitation is that participants may also have used over-the-counter medications, thereby underestimating excessive polypharmacy in our sample. We should acknowledge the limits of using pharmaceutical services database in the sense that obtaining a medication (by filling a prescription and purchasing it) does not necessarily prove actual use or consumption of the medication. Also, the year of survey completion (index date) was not related to a significant event in the patient's life trajectory (e.g., diagnosis). As a result, the trajectory model presented in this study yields a random picture of a part of participants' life span. It would be interesting in future studies to examine whether longer follow-up periods could capture more variations in patterns of excessive polypharmacy. TorSaDe Cohort's advantages clearly outweigh its disadvantages since, to our knowledge, no pain-specific Canadian data source outside tertiary care settings links self-reported data of thousands of patients with longitudinal health administrative data. Confounding was controlled through multivariable analysis in a large sample of participants and with a large variety of potential confounders. Although analyses were adjusted for comorbidities, benzodiazepines, and opioids use, not all classes of medications were accounted for. Finally, we cannot rule out the possibility of social desirability bias for certain variables such as alcohol consumption, smoking or physical activity.

## Conclusion

This project carried out in a large representative sample of people living with CP, highlighted that these individuals do not have the same chances of experiencing excessive polypharmacy according to different sociodemographic characteristics, pain-related variables, general health and lifestyle profile variables, and healthcare variables. Given the potential risks associated with excessive polypharmacy, it is important for clinicians to be able to identify these at-risk individuals that should be prioritized for a structured review of their medications. Nevertheless, polypharmacy can be rational (21, 34), by addressing pain through different mechanisms of action, in addition to comfort medications and for managing other diseases. Pharmacotherapy should therefore be approached by weighing the risks and benefits for each patient (personalized approach), in collaboration between the prescribers, community pharmacists, and other clinicians responsible for providing patient care and services.

## Data Availability

The data analyzed in this study is subject to the following licenses/restrictions: Our dataset, the TorSaDE Cohort data that links Statistics Canada's Canadian Community Health Survey (CCHS) data and Quebec Health Ministry data, is not publicly available. Access must be granted by the Institut de la statistique du Québec (ISQ) (data access authority). Programming codes can be obtained directly from the corresponding author. Requests to access these datasets should be directed to Institut de la statistique du Québec (ISQ), sad@stat.gouv.qc.ca.

## References

[1] Treede R-D Rief W Barke A Aziz Q Bennett MI Benoliel R Chronic pain as a symptom or a disease: the iasp classification of chronic pain for the international classification of diseases (Icd-11). Pain. (2019) 160(1):19–27. 10.1097/j.pain.0000000000001384 30586067

[2] Andrew R Derry S Taylor RS Straube S Phillips CJ. The costs and consequences of adequately managed chronic non-cancer pain and chronic neuropathic pain. Pain Pract. (2014) 14(1):79–94. 10.1111/papr.12050 23464879

[3] Zuccaro SM Vellucci R Sarzi-Puttini P Cherubino P Labianca R Fornasari D. Barriers to pain management: focus on opioid therapy. Clin Drug Investig. (2012) 32(1):11–9. 10.2165/11630040-000000000-00000 23389872

[4] MacDonald NE Flegel K Hebert PC Stanbrook MB. Better management of chronic pain care for all. CMAJ. (2011) 183(16):1815. 10.1503/cmaj.111065 21844104 PMC3216428

[5] Kingma EM Rosmalen JG. The power of longitudinal population-based studies for investigating the etiology of chronic widespread pain. Pain. (2012) 153(12):2305–6. 10.1016/j.pain.2012.09.001 23009898

[6] Sessle BJ. The pain crisis: what it is and what can be done. Pain Res Treat. (2012) 2012(703947):1–6. 10.1155/2012/703947 PMC3459223 23050138

[7] Kress HG Aldington D Alon E Coaccioli S Collett B Coluzzi F A holistic approach to chronic pain management that involves all stakeholders: change is needed. Curr Med Res Opin. (2015) 31(9):1743–54. 10.1185/03007995.2015.1072088 26172982

[8] Campbell M Hudspith M Anderson M Choinière M El-Gabalawy H Laliberté J Chronic Pain in Canada: Laying a Foundation for Action. Ottawa: Report by the Canadian Pain Task Force—Health Canada (2019).

[9] Campbell M Hudspith M Choinière M El-Gabalawy H Laliberté J Sangster M Working Together to Better Understand, Prevent and, Manage Chronic Pain: What We Heard. Ottawa: Report by the Canadian Pain Task Force—Health Canada (2020).

[10] Andersson HI Ejlertsson G Leden I Schersten B. Impact of chronic pain on health care seeking, self care, and medication. Results from a population-based Swedish study. J Epidemiol Community Health. (1999) 53(8):503–9. 10.1136/jech.53.8.503 10562870 PMC1756941

[11] Toblin RL Mack KA Perveen G Paulozzi LJ. A population-based survey of chronic pain and its treatment with prescription drugs. Pain. (2011) 152(6):1249–55. 10.1016/j.pain.2010.12.036 21397401

[12] Choinière M Dion D Peng P Banner R Barton PM Boulanger A The Canadian stop-pain project–part 1: who are the patients on the waitlists of multidisciplinary pain treatment facilities? Can J Anaesth. (2010) 57(6):539–48. 10.1007/s12630-010-9305-5 20393821

[13] Nguefack HL N Pagé M G Guénette L Blais L Diallo M Godbout-Parent M Gender differences in medication adverse effects experienced by people living with chronic pain. Front Pain Res. (2022) 3:830153. 10.3389/fpain.2022.830153 PMC9128021 35620635

[14] Chou R Hartung D Turner J Blazina I Chan B Levander X Opioid Treatments for Chronic Pain. Rockville (MD): Agency for Healthcare Research and Quality (2020). 32338848

[15] McDonagh MS Selph SS Buckley DI Holmes RS Mauer K Ramirez S Nonopioid Pharmacologic Treatments for Chronic Pain. Rockville (MD): Agency for Healthcare Research and Quality (2020). 32338847

[16] McQueenie R Jani BD Siebert S McLoone P McCowan C Macdonald S Prevalence of chronic pain in ltcs and multimorbidity: a cross-sectional study using UK biobank. J Multimorb Comorb. (2021) 11:26335565211005870. 10.1177/26335565211005870 35004337 PMC8728767

[17] Ferrari A Baraldi C Licata M Rustichelli C. Polypharmacy among headache patients: a cross-sectional study. CNS Drugs. (2018) 32(6):567–78. 10.1007/s40263-018-0522-8 29752625 PMC6061427

[18] Zahlan G De Clifford-Faugère G Nguena Nguefack HL Guénette L Pagé MG Blais L Polypharmacy and excessive polypharmacy among persons living with chronic pain: a cross-sectional study on the prevalence and associated factors. J Pain Res. (2023) 16:3085–100. 10.2147/JPR.S411451 37719270 PMC10505027

[19] Giummarra MJ Gibson SJ Allen AR Pichler AS Arnold CA. Polypharmacy and chronic pain: harm exposure is not all about the opioids. Pain Med. (2015) 16(3):472–9. 10.1111/pme.12586 25280054

[20] Thompson W Farrell B. Deprescribing: what is it and what does the evidence tell US? Can J Hosp Pharm. (2013) 66(3):201. 10.4212/cjhp.v66i3.1261 23814291 PMC3694945

[21] Masnoon N Shakib S Kalisch-Ellett L Caughey GE. What is polypharmacy? A systematic review of definitions. BMC Geriatr. (2017) 17(1):230. 10.1186/s12877-017-0621-2 29017448 PMC5635569

[22] Pazan F Wehling M. Polypharmacy in older adults: a narrative review of definitions, epidemiology and consequences. Eur Geriatr Med. (2021) 12(3):443–52. 10.1007/s41999-021-00479-3 33694123 PMC8149355

[23] World Health Organization. Medication Safety in Polypharmacy. Geneva: World Health Organization (2019).

[24] Jyrkka J Enlund H Korhonen MJ Sulkava R Hartikainen S. Patterns of drug use and factors associated with polypharmacy and excessive polypharmacy in elderly persons: results of the kuopio 75+ study: a cross-sectional analysis. Drugs Aging. (2009) 26(6):493–503. 10.2165/00002512-200926060-00006 19591524

[25] Onder G Liperoti R Fialova D Topinkova E Tosato M Danese P Polypharmacy in nursing home in Europe: results from the shelter study. J Gerontol A Biol Sci Med Sci. (2012) 67(6):698–704. 10.1093/gerona/glr233 22219520

[26] Carmona-Torres JM Cobo-Cuenca AI Recio-Andrade B Laredo-Aguilera JA Martins MM Rodríguez-Borrego MA. Prevalence and factors associated with polypharmacy in the older people: 2006–2014. J Clin Nurs. (2018) 27(15–16):2942–52. 10.1111/jocn.14371 29603814

[27] Chang TI Park H Kim DW Jeon EK Rhee CM Kalantar-Zadeh K Polypharmacy, hospitalization, and mortality risk: a nationwide cohort study. Sci Rep. (2020) 10(1):1–9. 10.1038/s41598-019-56847-4 33144598 PMC7609640

[28] Shi S Mörike K Klotz U. The clinical implications of ageing for rational drug therapy. Eur J Clin Pharmacol. (2008) 64(2):183–99. 10.1007/s00228-007-0422-1 18180915

[29] Nunnari P Ceccarelli G Ladiana N Notaro P. Prescribing cascades and medications most frequently involved in pain therapy: a review. Eur Rev Med Pharmacol Sci. (2021) 25(2):1034–41. 10.26355/eurrev_202101_24673 33577059

[30] Leelakanok N Holcombe AL Lund BC Gu X Schweizer ML. Association between polypharmacy and death: a systematic review and meta-analysis. J Am Pharm Assoc (2003). (2017) 57(6):729–38.e10. 10.1016/j.japh.2017.06.002 28784299

[31] Gosselin M Talbot D Simard M Chiu YM Mésidor M Boiteau V Classifying polypharmacy according to pharmacotherapeutic and clinical risks in older adults: a latent class analysis in Quebec, Canada. Drugs Aging. (2023) 40(6):573–83. 10.1007/s40266-023-01028-2 37149556

[32] Palapinyo S Methaneethorn J Leelakanok N. Association between polypharmacy and depression: a systematic review and meta-analysis. J Pharm Pract Res. (2021) 51(4):280–99. 10.1002/jppr.1749

[33] Schenker Y Park SY Jeong K Pruskowski J Kavalieratos D Resick J Associations between polypharmacy, symptom burden, and quality of life in patients with advanced, life-limiting illness. J Gen Intern Med. (2019) 34(4):559–66. 10.1007/s11606-019-04837-7 30719645 PMC6445911

[34] Kingsbury SJ Yi D Simpson GM. Psychopharmacology: rational and irrational polypharmacy. Psychiatr Serv. (2001) 52(8):1033–6. 10.1176/appi.ps.52.8.1033 11474046

[35] Comerci G J Katzman J Duhigg D. Controlling the swing of the opioid Pendulum. N Engl J Med. (2018) 378(8):691–3. 10.1056/NEJMp1713159 29466151

[36] Brandt J Shearer B Morgan SG. Prescription drug coverage in Canada: a review of the economic, policy and political considerations for universal pharmacare. J Pharm Policy Pract. (2018) 11:28. 10.1186/s40545-018-0154-x 30443371 PMC6220568

[37] Prendergast C Flood M Murry LT Clyne B Fahey T Moriarty F. Prescribing differences among older adults with differing health cover and socioeconomic Status: a cohort study. BMC Geriatr. (2023) 23(1):755. 10.1186/s12877-023-04441-9 37978448 PMC10656928

[38] Nguena Nguefack HL Pagé MG Katz J Choinière M Vanasse A Dorais M Trajectory modelling techniques useful to epidemiological research: a comparative narrative review of approaches. Clin Epidemiol. (2020) 12:1205–22. 10.2147/CLEP.S265287 33154677 PMC7608582

[39] Carr E Federman A Dzahini O Dobson RJ Bendayan R. A multidimensional measure of polypharmacy for older adults using the health and retirement study. Sci Rep. (2021) 11(1):8783. 10.1038/s41598-021-86331-x 33888728 PMC8062687

[40] Vanasse A Chiu YM Courteau J Dorais M Bartlett G Zawaly K Cohort profile: the care trajectories—enriched data (torsade) cohort. Int J Epidemiol. (2021) 50(4):1066-h. 10.1093/ije/dyaa167 33236074 PMC8407868

[41] Lacasse A Cauvier Charest E Dault R Cloutier A-M Choinière M Blais L Validity of algorithms for identification of individuals suffering from chronic noncancer pain in administrative databases: a systematic review. Pain Med. (2020) 21(9):1825–39. 10.1093/pm/pnaa004 32142130 PMC7553015

[42] Lacasse A Ware MA Dorais M Lanctôt H Choinière M. Is the Quebec provincial administrative database a valid source for research on chronic non-cancer pain? Pharmacoepidemiol Drug Saf. (2015) 24(9):980–90. 10.1002/pds.3820 26105572

[43] Statistics Canada. Canadian Community Health Survey—annual Component (Cchs)—detailed Information for 2012. Ottawa: Statistics Canada (2012). Available online at: http://www23.statcan.gc.ca/imdb/p2SV.pl?Function=getSurvey&Id=135927 (cited April 15, 2017).

[44] Raina P Bonnett B Waltner-Toews D Woodward C Abernathy T. How reliable are selected scales from population-based health surveys? An analysis among seniors. Can J Public Health. (1999) 90(1):60–4. 10.1007/BF03404102 10910569 PMC6979892

[45] Sanmartin C Decady Y Trudeau R Dasylva A Tjepkema M Fines P Linking the Canadian community health survey and the Canadian mortality database: an enhanced data source for the study of mortality. Health Rep. (2016) 27(12):10–8. 28002578

[46] Régie de l'assurance maladie du Québec. La Régie De L'assurance Maladie Du Québec Québec: Gouvernement du Québec. (2017). Available online at: http://www.ramq.gouv.qc.ca/fr/regie/Pages/mission.aspx (cited March 19, 2017).

[47] Régie de l'assurance maladie du Québec. Rapport Annuel De Gestion 2017–2018 Québec, Qc: Régie de l'assurance maladie du Québec, Gouvernement du Québec. (2018). Available online at: http://www.ramq.gouv.qc.ca/SiteCollectionDocuments/citoyens/fr/rapports/rappann1718.pdf (cited September 3, 2019).

[48] Tamblyn R Lavoie G Petrella L Monette J. The use of prescription claims databases in pharmacoepidemiological research: the accuracy and comprehensiveness of the prescription claims database in Quebec. J Clin Epidemiol. (1995) 48(8):999–1009. 10.1016/0895-4356(94)00234-H 7775999

[49] Bouhassira D Lanteri-Minet M Attal N Laurent B Touboul C. Prevalence of chronic pain with neuropathic characteristics in the general population. Pain. (2008) 136(3):380–7. 10.1016/j.pain.2007.08.013 17888574

[50] Boulanger A Clark AJ Squire P Cui E Horbay GL. Chronic pain in Canada: have we improved our management of chronic noncancer pain? Pain Res Manag. (2007) 12(1):39–47. 10.1155/2007/762180 17372633 PMC2670724

[51] Moulin DE Clark AJ Speechley M Morley-Forster PK. Chronic pain in Canada–prevalence, treatment, impact and the role of opioid analgesia. Pain Res Manag. (2002) 7(4):179–84. 10.1155/2002/323085 12518174

[52] Reitsma M Tranmer JE Buchanan DM VanDenKerkhof EG. The epidemiology of chronic pain in Canadian men and women between 1994 and 2007: longitudinal results of the national population health survey. Pain Res Manag. (2012) 17(3):166–72. 10.1155/2012/875924 22606681 PMC3401087

[53] Ramage-Morin PL Gilmour H. Chronic pain at ages 12 to 44. Health Rep. (2010) 21(4):53–61. 21269012

[54] Reitsma ML Tranmer JE Buchanan DM Vandenkerkhof EG. The prevalence of chronic pain and pain-related interference in the Canadian population from 1994 to 2008. Chronic Dis Inj Can. (2011) 31(4):157–64. 10.24095/hpcdp.31.4.04 21978639

[55] Gilmour H. Chronic pain, activity restriction and flourishing mental health. Health Rep. (2015) 26(1):15–22. 25606984

[56] Ramage-Morin PL. Chronic pain in Canadian seniors. Health Rep. (2008) 19(1):37–52. 18457210

[57] Hogan ME Taddio A Katz J Shah V Krahn M. Health utilities in people with chronic pain using a population-level survey and linked health care administrative data. Pain. (2017) 158(3):408–16. 10.1097/j.pain.0000000000000776 27902568

[58] Hogan ME Taddio A Katz J Shah V Krahn M. Incremental health care costs for chronic pain in Ontario, Canada: a population-based matched cohort study of adolescents and adults using administrative data. Pain. (2016) 157(8):1626–33. 10.1097/j.pain.0000000000000561 26989805

[59] Hovstadius B Astrand B Petersson G. Dispensed drugs and multiple medications in the Swedish population: an individual-based register study. BMC Clin Pharmacol. (2009) 9:11. 10.1186/1472-6904-9-11 19473486 PMC2696422

[60] Van der Heyden J Berete F Renard F Vanoverloop J Devleesschauwer B De Ridder K Assessing polypharmacy in the older population: comparison of a self-reported and prescription based method. Pharmacoepidemiol Drug Saf. (2021) 12:1716–26. 10.1002/pds.5321 34212435

[61] Jones BL Nagin DS. Advances in group-based trajectory modeling and an sas procedure for estimating them. Sociol Methods Res. (2007) 35(4):542–71. 10.1177/0049124106292364

[62] Nagin DS Odgers C. Group-based trajectory modeling in clinical research. Annu Rev Clin Psychol. (2010) 6:109–38. 10.1146/annurev.clinpsy.121208.131413 20192788

[63] Jones BL Nagin DS Roeder K. A sas procedure based on mixture models for estimating developmental trajectories. Sociol Methods Res. (2001) 29(3):374–93. 10.1177/0049124101029003005

[64] Nagin DS. Analyzing developmental trajectories: a semiparametric, group-based approach. Psychosoc Med. (1999) 4(2):139–57. 10.1037/1082-989X.4.2.139

[65] Andersen RM. Revisiting the behavioral model and access to medical care: does it matter? J Health Soc Behav. (1995) 36(1):1–10. 10.2307/2137284 7738325

[66] Babitsch B Gohl D von Lengerke T. Re-revisiting Andersen’s behavioral model of health services use: a systematic review of studies from 1998 to 2011. Psychosoc Med. (2012) 9:Doc11. 10.3205/psm000089 23133505 PMC3488807

[67] Gamboa CM Colantonio LD Brown TM Carson AP Safford MM. Race-sex differences in statin use and low-density lipoprotein cholesterol control among people with diabetes mellitus in the reasons for geographic and racial differences in stroke study. J Am Heart Assoc. (2017) 6(5):e004264. 10.1161/JAHA.116.004264 28490523 PMC5524054

[68] Simard M Sirois C Candas B. Validation of the combined comorbidity Index of charlson and elixhauser to predict 30-day mortality across Icd-9 and Icd-10. Med Care. (2018) 56(5):441–7. 10.1097/mlr.0000000000000905 29578951

[69] CIHR. How to Integrate Sex and Gender into Research Ottawa: Canadian Institutes of Health Research (2023). Available online at: http://www.cihr-irsc.gc.ca/e/50836.html (cited March, 2023).

[70] Sourial N Vedel I Le Berre M Schuster T. Testing group differences for confounder selection in nonrandomized studies: flawed practice. CMAJ. (2019) 191(43):E1189–E93. 10.1503/cmaj.190085 31659059 PMC6821495

[71] Katz MH. Multivariable Analysis: A Practical Guide for Clinicians and Public Health Researchers. New York: Cambridge University Press (2011).

[72] Vatcheva KP Lee M McCormick JB Rahbar MH. Multicollinearity in regression analyses conducted in epidemiologic studies. Epidemiology. (2016) 6(2):227. 10.4172/2161-1165.1000227 27274911 PMC4888898

[73] Berndt MC Schütz HW. Polymedication and medication compliance in patients with chronic non-malignant pain. Pain. (1993) 52(3):331–9. 10.1016/0304-3959(93)90167-n 8460051

[74] Ramage-Morin PL. Medication use among senior Canadians. Health Rep. (2009) 20(1):37–44. 19388367

[75] Schneider J Algharably EAE Budnick A Wenzel A Dräger D Kreutz R. High prevalence of multimorbidity and polypharmacy in elderly patients with chronic pain receiving home care are associated with multiple medication-related problems. Front Pharmacol. (2021) 12:686990. 10.3389/fphar.2021.686990 34168565 PMC8217758

[76] Siebenhuener K Eschmann E Kienast A Schneider D Minder CE Saller R Chronic pain: how challenging are ddis in the analgesic treatment of inpatients with multiple chronic conditions? PLoS One. (2017) 12(1):e0168987. 10.1371/journal.pone.0168987 28046033 PMC5207693

[77] Taylor R Jr. Pergolizzi VJJ Puenpatom RA Summers KH. Economic implications of potential drug-drug interactions in chronic pain patients. Expert Rev Pharmacoecon Outcomes Res. (2013) 13(6):725–34. 10.1586/14737167.2013.851006 24219048

[78] Antony M Lee KY Bertram CT Abarca J Rehfeld RA Malone DC Gender and age differences in medications dispensed from a national chain drugstore. J Womens Health. (2008) 17(5):735–43. 10.1089/jwh.2007.0731 18537477

[79] Stock SAK Stollenwerk B Redaelli M Civello D Lauterbach KW. Sex differences in treatment patterns of six chronic diseases: an analysis from the German statutory health insurance. J Womens Health. (2008) 17(3):343–54. 10.1089/jwh.2007.0422 18338965

[80] Rianon N Knell ME Agbor-Bawa W Thelen J Burkhardt C Rasu RS. Persistent nonmalignant pain management using nonsteroidal anti-inflammatory drugs in older patients and use of inappropriate adjuvant medications. Drug Healthc Patient Saf. (2015) 7:43–50. 10.2147/dhps.S67425 25678818 PMC4319679

[81] Hales CM Martin CB Gu Q. Prevalence of prescription pain medication use among adults: United States, 2015–2018. NCHS Data Brief. (2020) (369):1–8. 32600518

[82] Haider SI Johnell K Thorslund M Fastbom J. Trends in polypharmacy and potential drug-drug interactions across educational groups in elderly patients in Sweden for the period 1992–2002. Int J Clin Pharmacol Ther. (2007) 45(12):643–53. 10.5414/cpp45643 18184532

[83] Delara M Murray L Jafari B Bahji A Goodarzi Z Kirkham J Prevalence and factors associated with polypharmacy: a systematic review and meta-analysis. BMC Geriatr. (2022) 22(1):601. 10.1186/s12877-022-03279-x 35854209 PMC9297624

[84] Veehof L Stewart R Haaijer-Ruskamp F Jong BM. The development of polypharmacy. A longitudinal study. Fam Pract. (2000) 17(3):261–7. 10.1093/fampra/17.3.261 10846147

[85] Hajjar ER Cafiero AC Hanlon JT. Polypharmacy in elderly patients. Am J Geriatr Pharmacother. (2007) 5(4):345–51. 10.1016/j.amjopharm.2007.12.002 18179993

[86] Rudnicka E Napierała P Podfigurna A Męczekalski B Smolarczyk R Grymowicz M. The world health organization (who) approach to healthy ageing. Maturitas. (2020) 139:6–11. 10.1016/j.maturitas.2020.05.018 32747042 PMC7250103

[87] Canadian Institutes of Health Research. Cihr Institute of Aging Strategic Plan 2019–2021: Living Longer, Living Better. Ottawa: Government of Canada (2023).

[88] World Health Organization. Ageing and Health. Geneva: WHO (2023). Available online at: https://www.who.int/news-room/fact-sheets/detail/ageing-and-health (cited September, 2023).

[89] Burke A Kuo T Harvey R Wang J. An international comparison of attitudes toward traditional and modern medicine in a Chinese and an American clinic setting. Evid Based Complement Alternat Med. (2011) 2011:204137. 10.1093/ecam/nen065 18955368 PMC3136226

[90] Rojas P Jung-Cook H Ruiz-Sánchez E Rojas-Tomé IS Rojas C López-Ramírez AM Historical aspects of herbal use and comparison of current regulations of herbal products between Mexico, Canada and the United States of America. Int J Environ Res Public Health. (2022) 19(23):15690. 10.3390/ijerph192315690 36497761 PMC9740500

[91] Huiskes VJ Burger DM van den Ende CH van den Bemt BJ. Effectiveness of medication review: a systematic review and meta-analysis of randomized controlled trials. BMC Fam Pract. (2017) 18(1):5. 10.1186/s12875-016-0577-x 28095780 PMC5240219

[92] Campbell F Hudspith M Choinière M El-Gabalawy H Laliberté J Sangster M An Action Plan for Pain in Canada. *Report by the Canadian Pain Task Force—Health Canada*. (2021).

