# Supplementary material for: Unpacking excessive polypharmacy patterns among individuals living with chronic pain in Quebec: a longitudinal study
Source: Front Pain Res (Lausanne). 2025 Feb 21;6:1512878. doi: 10.3389/fpain.2025.1512878 (PMC11885514; doi:10.3389/fpain.2025.1512878)
Supplement: Supplementary file 1 [file Datasheet1.pdf]

Supplementary material 1 - Model fit indices tables

Table legend:

[1] Trajectory shape: 1 = linear (straight line); 2 = linear + quadratic (u-shaped curve/parabola).

[2] BIC: Bayesian information criterion.

Bold text: Model which best fits the data and respected all criteria (lowest BIC absolute value among trajectory groups that respected the 5% criteria).

* One or more groups in the model had less than 5% of participants.

** Lowest BIC but not the most clinically interpretable model.

Model fit indices for trajectories of polypharmacy in the whole study sample

Number of trajectories	Trajectory shape [1]	BIC [2] (n=9156)
1	1	-50448.47
1	2	-50452.72
2	11	-22750.35
2	21	-22749.67
2	12	-22747.68
2	22	-22746.34
3	111	-20341.26
3	121	-20343.83
3	211	-20343.83
3	221	-20348.39
3	112	-20333.14
3	122	-20335.71
3	212	-20957.95
3	222	-20340.23
4	1121	-20070.33
**4**	**1122**	**-20063.09**
4	1211	-20135.79

4	1221	-20073.53
4	2121	-20145.63
4	2211	-20148.99
4	2221	-20661.45
4	1112	-20117.93
4	1212	-20121.95
4	1222	-20065.78
4	2112	-20072.50
4	2122	-20147.40
4	2212	-20127.97
4	2222	-20067.52
5	11111*	-19982.86
5	11121*	-19985.49
5	11211	-20064.54
5	11221	-20065.74
5	12111*	-19985.56
5	12121*	-19988.19
5	12211*	-19988.19

Model fit indices for trajectories of polypharmacy in males

Number of trajectories	Trajectory shape [1]	BIC [2] (n=3248)
1	1	-16079.73
1	2	-16083.44
2	11	-6874.96
2	12	-6875.68
2	21	-6878.00
2	22	-6878.53
3	111	-6175.39
3	112	-6175.61

3	121	-6179.33
3	211	-6274.60
3	122	-6179.54
3	212	-6179.54
3	221	-6283.31
3	222	-6183.58
4	1111*	-6119.45
**4**	**1112**	**-6121.17**
4	1121*	-6091.56
4	1122*	-6120.62
4	1211*	-6123.49
4	2111*	-6123.49
4	1212*	-6103.97
4	1221*	-6126.14
4	2121*	-6092.59
4	2112*	-6126.46
4	2211	-6192.15
4	1222*	-6110.94
4	2122	Convergence trouble
4	2212*	-6129.12
4	2221*	-6096.10
4	2222*	-6126.46

Model fit indices for trajectories of polypharmacy in females

Number of trajectories	Trajectory shape [1]	BIC [2] (n=5908)
1	1	-34207.17
1	2	-34211.43
2	11	-15849.95
2	12	-15850.50

2	21	-15850.71
2	22	-15850.84
3	111	-14160.58
3	112	-14155.98
3	121	-14194.57
3	211	-14161.89
3	122	-14157.28
3	212	-14157.28
3	221	-14166.23
3	222	-14161.57
4	1111	-13972.80
4	1112*	-14003.22
4	1121	-13971.45
4	1122	-13974.77
**4**	**1211**	**-13921.97**
4	1212	-14001.87
4	1221	-13983.63
4	1222	-13994.96
4	2222*	Convergence trouble
4	2221	-14036.08
4	2212*	-13980.23
4	2111*	-14036.46
4	2112	-13976.20
4	2121*	-14037.40
4	2122	-14026.20
4	2211	-13978.12
5	22222	Convergence trouble
5	11111**	-13967.67
5	12111**	-13972.03

Supplementary material 2 – Profile of participants in each trajectory.

Statistically significant differences were found between groups for the following variables:

Sociodemographic variables: Age, sex at birth, white self-identified race, country of birth, education level, relationship status, annual household income, number of people in the household, living in a remote region, living in a rural area).

Pain related variables: Pain intensity, activities prevented by pain, self-reported back pain, self-reported arthritis, opioids use 30 days after survey completion, benzodiazepines use 30 days after survey completion.

General health and lifestyle profile variables: Combined comorbidity index of Charlson and Elixhauser, perceived mental health, perceived general health, alcohol consumption in the past 12 months, physical activity, lifestyle.

Healthcare variables: Visiting a pain clinic in the year before survey completion, reporting having a regular physician, and the past-year number of hospitalizations, visits to a family physician, visits to a specialist, emergency room visits, prescribers consulted, and different physicians consulted.

**Characteristics** (n = 9156)	**No excessive polypharmacy** (trajectory2) **n = 6943**	**Sometimes in excessive polypharmacy** (trajectory1) **n=697**	**Often in excessive polypharmacy** (trajectory3) **n=536**	**Always in excessive polypharmacy** (trajectory4) **n=980**
**Sociodemographic profile**	**75.83%**	**7.61%**	**5.85%**	**10.70%**
**Age (years)** †**				
18-64	3526 (50.78%)	236 (33.86%)	134 (25.00%)	299 (30.51%)
65-79	2683 (38.64%)	326 (46.77%)	267 (49.81%)	460 (46.94%)
≥ 80	734 (10.57%)	135 (19.37%)	135 (25.19%)	221 (22.55%)
**Sex at birth****				
Females	4349 (62.64%)	498 (71.45%)	379 (70.71%)	682 (69.59%)
Males	2594 (37.36%)	199 (28.55%)	157 (29.29%)	298 (30.41%)
**White self-identified race****				
Yes	6447 (92.86%)	662 (94.98%)	510 (95.15%)	944 (96.33%)
No	496 (7.14%)	35 (5.02%)	26 (4.85%)	36 (3.67%)
**Country of birth****				
Canada	6409 (92.34%)	652 (93.68%)	499 (93.10%)	943 (96.22%)
Other	532 (7.66%)	44 (6.32%)	37 (6.90%)	37 (3.78%)
**Education level****				
No secondary diploma	2585 (37.70%)	345 (50.15%)	276 (52.57%)	545 (56.65%)
Secondary diploma	917 (13.38%)	78 (11.34%)	70 (13.33%)	106 (11.02%)
College diploma/ Registered apprenticeship/ other trades certificate or diploma	2496 (36.41%)	196 (28.49%)	129 (24.57%)	256 (26.61%)
University diploma	858 (12.51%)	69 (10.03%)	50 (9.52%)	55 (5.72%)
**Relationship status ****				
In a relationship	3051 (43.98%)	257 (36.87%)	200 (37.31%)	339 (34.59%)
Not in a relationship	3887 (56.02%)	440 (63.13%)	336 (62.69%)	641 (65.41%)
**Annual household income (Can$)** †**				
< 20,000	2097 (30.20%)	267 (38.31%)	228 (42.54%)	491 (50.10%)
20,000 -39,999	2657 (38.27%)	273 (39.17%)	208 (38.81%)	344 (35.10%)
40,000 – 59,999	1220 (17.57%)	96 (13.77%)	66 (12.31%)	86 (8.78%)

≥ 60,000	969 (13.96%)	61 (8.75%)	34 (6.34%)	59 (6.02%)
**Number of people in the household**** – mean ± SD	1.75 ± 0.96	1.53 ± 0.74	1.55 ± 0.83	1.59 ± 2.45
**Living in a remote region****				
Yes	1818 (26.18%)	190 (27.26%)	149 (27.80%)	291 (29.69%)
No	5125 (73.82%)	507 (72.74%)	387 (72.20%)	689 (70.31%)
**Living in a rural area****				
Yes	2254 (32.46%)	207 (29.70%)	143 (26.68%)	294 (30.00%)
No	4689 (67.54%)	490 (70.30%)	393 (73.32%)	686 (70.00%)
**Pain symptoms**				
**Pain intensity****				
Mild	1581 (22.98%)	86 (12.43%)	69 (13.09%)	100 (10.36%)
Moderate or severe	5298 (77.02%)	606 (87.57%)	458 (86.91%)	865 (89.64%)
**Activities prevented by pain or discomfort****				
None or a few	5000 (72.37%)	403 (58.15%)	267 (50.38%)	449 (46.05%)
Some or most	1909 (27.63%)	290 (41.85%)	263 (49.62%)	526 (53.95%)
**Self-reported back pain (except fibromyalgia and arthritis)****				
Yes	2870 (41.47%)	315 (45.59%)	223 (41.76%)	501 (51.23%)
**Self-reported arthritis (except fibromyalgia)****				
Yes	3021 (43.76%)	424 (61.18%)	352 (65.92%)	638 (65.44%)
**Opioids use 30 days after survey completion****				
Yes	313 (4.51%)	84 (12.05%)	70 (13.06%)	223 (22.76%)
**Benzodiazepines use 30 days after survey completion****				
Yes	1391 (20.03%)	328 (47.06%)	248 (46.27%)	594 (60.61%)
**General health and lifestyle profile**				
**Combined comorbidity index of Charlson and Elixhauser****– mean ± SD	0.39 ± 1.19	1.07 ± 2.20	1.24 ± 2.24	1.59 ± 2.45
**Perceived general health****				

Excellent, very good or good	4677 (67.48%)	279 (40.14%)	215 (40.11%)	267 (27.27%)
Fair or bad	2254 (32.52%)	416 (59.86%)	321 (59.89%)	712 (72.73%)
**Perceived mental health****				
Excellent, very good or good	6066 (90.32%)	563 (84.79%)	429 (85.80%)	750 (82.78%)
Fair or bad	650 (9.68%)	101 (15.21%)	71 (14.20%)	156 (17.22%)
**Alcohol consumption in the past 12 months****				
Regular	3949 (57.11%)	263 (37.90%)	214 (40.00%)	275 (28.21%)
Occasional or has not drunk	2966 (42.89%)	431 (62.10%)	321 (60.00%)	700 (71.79%)
**Smoking**				
Regular	1587 (22.86%)	150 (21.52%)	102 (19.03%)	210 (21.43%)
Occasional or never	5354 (77.14%)	547 (78.48%)	434 (80.97%)	770 (78.57%)
**Physical activity ****				
Regular	3767 (56.01%)	282 (42.41%)	166 (33.13%)	279 (30.79%)
Occasional or rare	2958 (43.99%)	383 (57.59%)	335 (66.87%)	627 (69.21%)
**Lifestyle ****				
Active or moderately active	2710 (40.30%)	183 (27.52%)	98 (19.56%)	174 (19.21%)
Inactive	4015 (59.70%)	482 (72.48%)	403 (80.44%)	732 (80.79%)
**Health care**				
**Visiting a pain clinic in the year before survey completion****				
Yes	103 (1.48%)	18 (2.58%)	16 (2.99%)	26 (2.65%)
**Having a family physician****				
Yes	5737 (82.63%)	650 (93.26%)	500 (93.28%)	927 (94.59%)
**Past-year number of hospitalizations**** – mean ± SD	0.19 ± 0.59	0.39 ± 0.82	0.47 ± 2.24	0.56 ± 0.98
**Past-year number of visits to a family physician**** – mean ± SD	3.05 ± 3.15	4.75 ± 5.34	4.51 ± 3.73	5.03 ± 3.87
**Past-year number of specialist visits**** – mean ± SD	3.15 ± 4.79	6.04 ± 8.65	6.67 ± 11.37	7.07 ± 12.40
**Past-year number of emergency room visits**** – mean ± SD	0.57 ± 1.24	0.91 ± 1.59	1.14 ± 1.90	1.40 ± 2.20

**Past-year number of prescribers consulted**** – mean ± SD	2.30 ± 1.77	3.74 ± 2.31	4.06 ± 2.59	4.57 ± 3.10
**Past-year number of different physicians consulted**** – mean ± SD	4.45 ± 4.04	7.09 ± 5.82	7.98 ± 6.64	8.79 ± 7.47

Table footnotes:

* Bivariate comparison p-value < 0.05 and ** p-value < 0.001 (Bonferroni correction: p-value<0.0016).

† Categories have been redesigned to comply with confidentiality rules.

The variable "Indigenous self-identification" had to be excluded because n<15.

Supplementary material 3 – Determinants of Trajectory Membership.

(reference "no polypharmacy", n=5766)

**Characteristics *** (n = 7644)	**Sometimes in excessive polypharmacy** (#trajectory1) **n=595**	**Often in excessive polypharmacy** (#trajectory3) **n=450**	**Always in excessive polypharmacy** (#trajectory4) **n=833**
**Sociodemographic profile**			
**Age (years)**	OR 1.03 95% CI 1.02 to 1.04	OR 1.04 95% CI 1.03 to 1.06	OR 1.04 95% CI 1.03 to 1.05
**Sex at birth**			
Females (vs. Males)	OR 1.5 95% CI 0.93 to 1.42	OR 1.09 95% CI 0.85 to 1.39	OR 0.96 95% CI 0.79 to 1.18
**White self-identified race**			
Yes (vs. No)	OR 1.27 95% CI 0.53 to 3.07	OR 0.91 95% CI 0.35 to 2.38	OR 1.61 95% CI 0.59 to 4.40
**Country of birth**			
Canada (vs. Other)	OR 1.08 95% CI 0.63 to 1.84	OR 1.82 95% CI 0.94 to 3.54	OR 2.60 95% CI 1.37 to 4.96
**Education level** (vs. No secondary diploma)			
Secondary diploma	OR 0.88 95% CI 0.66 to 1.19	OR 1.08 95% CI 0.78 to 1.50	OR 0.90 95% CI 0.68 to 1.21
College diploma/ Registered apprenticeship/ other trades certificate or diploma	OR 0.92 95% CI 0.74 to 1.15	OR 1.01 95% CI 0.78 to 1.30	OR 1.05 95% CI 0.85 to 1.30
University diploma	OR 1.00 95% CI 0.71 to 1.40	OR 1.16 95% CI 0.78 to 1.71	OR 0.76 95% CI 0.52 to 1.11
**Relationship status**			
In a relationship (vs. not in a relationship)	OR 0.94 95% CI 0.73 to 1.21	OR 1.05 95% CI 0.79 to 1.39	OR 1.21 95% CI 0.95 to 1.56
**Annual household income (Can$)** (vs. < 20,000)			
20,000 – 39,999	OR 1.01 95% CI 0.81 to 1.27	OR 0.66 95% CI 0.51 to 0.85	OR 0.60 95% CI 0.48 to 0.75
40,000 – 59,999	OR 1.09 95% CI 0.79 to 1.50	OR 0.57 95% CI 0.39 to 0.83	OR 0.45 95% CI 0.32 to 0.64
60,000 – 79,999	OR 1.14 95% CI 0.70 to 1.84	OR 0.65 95% CI 0.37 to 1.13	OR 0.45 95% CI 0.26 to 0.77
≥ 80,000	OR 0.88 95% CI 0.49 to 1.59	OR 0.37 95% CI 0.18 to 0.80	OR 0.64 95% CI 0.36 to 1.15
**Number of people in the household**	OR 0.87 95% CI 0.74 to 1.04	OR 1.02 95% CI 0.87 to 1.21	OR 0.92 95% CI 0.78 to 1.16
**Living in a remote region**			
Yes (vs. No)	OR 1.06 95% CI 0.85 to 1.32	OR 1.24 95% CI 0.96 to 1.59	OR 1.10 95% CI 0.89 to 1.36

**Living in an urban area**<br>Yes (vs. No)	OR 0.97 95% CI 0.79 to 1.20	OR 1.19 95% CI 0.93 to 1.52	OR 1.07 95% CI 0.87 to 1.31
**Pain symptoms**			
**Pain intensity**<br>Moderate or severe (vs. Mild)	OR 1.55 95% CI 1.18 to 2.05	OR 1.25 95% CI 0.92 to 1.70	OR 1.48 95% CI 1.12 to 1.96
**Activities prevented by pain or discomfort**<br>Some or most (vs. None or a few)	OR 1.18 95% CI 0.97 to 1.45	OR 1.57 95% CI 1.25 to 1.97	OR 1.52 95% CI 1.26 to 1.83
**Self-reported back pain (except fibromyalgia and arthritis)**<br>Yes	OR 1.08 95% CI 0.90 to 1.31	OR 0.93 95% CI 0.75 to 1.15	OR 1.25 95% CI 1.04 to 1.50
**Self-reported arthritis (except fibromyalgia)**<br>Yes	OR 1.30 95% CI 1.07 to 1.58	OR 1.39 95% CI 1.12 to 1.74	OR 1.41 95% CI 1.17 to 1.70
**Opioids use 30 days after survey completion**<br>Yes	OR 1.43 95% CI 1.05 to 1.94	OR 1.83 95% CI 1.32 to 2.53	OR 2.83 95% CI 2.20 to 3.63
**Benzodiazepines use 30 days after survey completion**<br>Yes (vs. No)	OR 2.05 95% CI 1.70 to 2.47	OR 1.95 95% CI 1.58 to 2.47	OR 3.04 95% CI 2.55 to 3.64
**General health and lifestyle profile**			
**Combined comorbidity index of Charlson and Elixhauser**	OR 1.09 95% CI 1.03 to 1.16	OR 1.10 95% CI 1.03 to 1.17	OR 1.12 95% CI 1.06 to 1.19
**Perceived general health**<br>Fair or bad (vs. Excellent, very good or good)	OR 1.90 95% CI 1.55 to 2.33	OR 1.65 95% CI 1.31 to 2.08	OR 2.41 95% CI 1.97 to 2.94
**Perceived mental health**<br>Fair or bad (vs. Excellent, very good or good)	OR 1.04 95% CI 0.79 to 1.37	OR 1.08 95% CI 0.79 to 1.47	OR 0.94 95% CI 0.73 to 1.21
**Alcohol consumption in the past 12 months** (vs. regular)<br>Occasional<br>Has not drunk	 OR 1.76 95% CI 1.40 to 2.23<br>OR 1.72 95% CI 1.38 to 2.15	 OR 1.21 95% CI 0.93 to 1.59<br>OR 1.27 95% CI 0.99 to 1.63	 OR 1.57 95% CI 1.24 to 1.99<br>OR 2.28 95% CI 1.85 to 2.80

**Smoking** (vs. regular)<br>Occasional<br>Never	 OR 0.60 95% CI 0.31 to 1.19<br>OR 0.98 95% CI 0.77 to 1.24	 OR 0.78 95% CI 0.37 to 1.65<br>OR 1.03 95% CI 0.77 to 1.36	 OR 0.77 95% CI 0.42 to 1.40<br>OR 1.18 95% CI 0.93 to 1.49
**Physical activity** (vs. regular)<br>Occasional<br>Rare	 OR 1.09 95% CI 0.79 to 1.51<br>OR 1.09 95% CI 0.84 to 1.42	 OR 1.17 95% CI 0.80 to 1.72<br>OR 1.49 95% CI 1.10 to 2.02	 OR 1.19 95% CI 0.85 to 1.66<br>OR 1.68 95% CI 1.29 to 2.19
**Lifestyle (PACDPAI)** (vs. active)<br>Moderately active<br>Inactive	 OR 0.89 95% CI 0.63 to 1.25<br>OR 1.01 95% CI 0.72 to 1.42	 OR 1.44 95% CI 0.89 to 2.34<br>OR 1.77 95% CI 1.09 to 2.87	 OR 1.01 95% CI 0.69 to 1.46<br>OR 1.12 95% CI 0.77 to 1.62
**Health care**			
**Visiting a pain clinic in the year before survey completion**<br>Yes (vs. No)	OR 0.97 95% CI 0.53 to 1.76	OR 0.96 95% CI 0.49 to 1.89	OR 0.91 95% CI 0.52 to 1.60
**Having a family physician**<br>Yes (vs. No)	OR 1.47 95% CI 1.01 to 1.93	OR 1.28 95% CI 0.84 to 1.93	OR 1.77 95% CI 1.20 to 2.61
**Past-year number of hospitalizations**	OR 0.93 95% CI 0.80 to 1.07	OR 0.88 95% CI 0.75 to 1.03	OR 0.89 95% CI 0.78 to 1.02
**Past-year number of visits to a family physician**	OR 1.05 95% CI 1.02 to 1.07	OR 1.02 95% CI 0.99 to 1.05	OR 1.04 95% CI 1.01 to 1.06
**Past-year number of specialist visits**	OR 1.02 95% CI 1.00 to 1.03	OR 1.02 95% CI 1.00 to 1.03	OR 1.02 95% CI 1.00 to 1.04
**Past-year number of emergency room visits**	OR 0.92 95% CI 0.85 to 0.99	OR 0.92 95% CI 0.85 to 1.00	OR 0.94 95% CI 0.88 to 1.00
**Past-year number of prescribers consulted**	OR 1.31 95% CI 1.24 to 1.38	OR 1.33 95% CI 1.25 to 1.41	OR 1.40 95% CI 1.33 to 1.48
**Past-year number of different physicians consulted**	OR 1.00 95% CI 0.97 to 1.03	OR 1.03 95% CI 0.10 to 1.06	OR 1.01 95% CI 0.98 to 1.04
